# Edge Structural Health Monitoring (E-SHM) Using Low-Power Wireless Sensing

**DOI:** 10.3390/s21206760

**Published:** 2021-10-12

**Authors:** Tadhg Buckley, Bidisha Ghosh, Vikram Pakrashi

**Affiliations:** 1 UCD Centre for Mechanics, Dynamical Systems and Risk Laboratory, School of Mechanical and Materials Engineering, University College Dublin, Dublin 4, Ireland; Tadhg.Buckley@ucdconnect.ie; 2 QUANT Group, Department of Civil, Structural and Environmental Engineering, Trinity College Dublin, Dublin 2, Ireland; bghosh@tcd.ie

**Keywords:** structural health monitoring, edge, long-range, microcontroller, IoT, damage sensitive feature, acceleration, cloud

## Abstract

Effective Structural Health Monitoring (SHM) often requires continuous monitoring to capture changes of features of interest in structures, which are often located far from power sources. A key challenge lies in continuous low-power data transmission from sensors. Despite significant developments in long-range, low-power telecommunication (e.g., LoRa NB-IoT), there are inadequate demonstrative benchmarks for low-power SHM. Damage detection is often based on monitoring features computed from acceleration signals where data are extensive due to the frequency of sampling (~100–500 Hz). Low-power, long-range telecommunications are restricted in both the size and frequency of data packets. However, microcontrollers are becoming more efficient, enabling local computing of damage-sensitive features. This paper demonstrates the implementation of an Edge-SHM framework through low-power, long-range, wireless, low-cost and off-the-shelf components. A bespoke setup is developed with a low-power MEM accelerometer and a microcontroller where frequency and time domain features are computed over set time intervals before sending them to a cloud platform. A cantilever beam excited by an electrodynamic shaker is monitored, where damage is introduced through the controlled loosening of bolts at the fixed boundary, thereby introducing rotation at its fixed end. The results demonstrate how an IoT-driven edge platform can benefit continuous monitoring.

## 1. Introduction

Implementing a monitoring system on full-scale structures is a complex and often expensive task, which requires both domain expertise and experience in the development of an end-to-end data pipeline, from sensor acquisition and communication to data analysis and inference [1,2,3].

Wired sensing systems are difficult to install, particularly for an operational structure, have large power consumption, are susceptible to disturbances and damage and have a high cost of implementation and maintenance [4]. In contrast, wireless systems are often quicker and easier to implement, are suitable for scaling across an infrastructure network and can be more than 10 times cheaper to deploy [5]. Long-term monitoring of an infrastructure network requires low-cost, wireless, battery-powered sensing devices. There are several challenges involved in the deployment of wireless sensor networks (WSN) for SHM. These include data storage and transmission, the requirement for low power and the cost and complexity of implementation. The primary data source used for SHM is acceleration with a sampling rate in the range of ~100–500 Hz. This sampling rate creates large volumes of data, particularly for multiple sensors recording on a structure. The data can either be stored locally or communicated to an offsite or cloud database for analysis. Local storage requires the collection of significant amounts of data that can only be analysed after being regathered. 

Communication protocols with high data throughput (e.g., 4G) have significant power demand and are not suitable for wireless monitoring applications. The lack of available long-term data monitoring data due to the difficulty in acquiring them from operational structures has led to researchers relying on numerical models and experimental structures. A small number of full-scale datasets, such as that from the controlled tests on the Z24 bridge [6], are available for the development of damage detection techniques. Alternative Low-Power Wide-Area Networks (LPWAN) [7] have been developed for wireless sensors applications for the Internet of Things (IoT). These include cellular-based technologies such as NarrowBand-IoT (NB-IoT) [8] and technologies such as SigFox and LoRa [9,10], which operate in the unlicensed scientific bands.

An end-to-end, long-range, low-power monitoring system consists of an edge device, a gateway and a cloud platform. The edge device or wireless sensor node consists of sensors, a microcontroller, a wireless transmission board and a battery-power module. They require basic functionality including (*i*) data *acquisition* from multiple sensors, (*ii*) scheduling and execution of *measurement* tasks, (*iii*) *storage* of measured data, (*iv*) on-board data *processing* and *analysis* (e.g., signal processing, feature extraction) and (*v*) *communication* and *networking* with the base station/gateway via a low-power communication protocol involving reliable transmission routing of data packets to the base station and receiving commands from the base station for reconfiguration. The gateway can be in the form of a private LoRa gateway, a public LoRaWAN gateway, or an NB-IoT mast. Data are transmitted from each gateway to a cloud platform for storage, analysis and visualisation. Once the gateway has received data from the edge nodes, it can then be transmitted to a cloud or offsite platform via Wi-Fi or 4G using standard IP protocol for the LoRa/LoRaWAN gateways. Data from the NB-IoT gateways are in the form of a Constrained Application Protocol (CoAP), and an intermediary port needs to be configured for a custom cloud setup to receive packets. Despite these advantages, LPWANs are restricted in terms of the size and rate of data packets that can be transmitted. Feature extraction at the edge also presents challenges due to the computational constraints of a low-power device.

There is a disconnect between the complex feature extraction and damage detection algorithms developed by SHM researchers and the type of data that can be obtained using low-power wireless sensor nodes. Modal feature extraction methods such as stochastic subspace identification [11] are often unsuitable for real-time wireless monitoring as they require multi-sensor datasets of raw acceleration from locations on the entire structure. 

Continuous remote wireless monitoring of a structure requires data compression or univariate feature extraction at the edge. Therefore, features must be pre-coded onto the edge sensor before deployment. Univariate, single-channel features extracted from the recorded vibrations can be categorised in time, frequency and time–frequency domains, with the latter two being associated with significant computational complexity, which results in processing requirements at the edge and therefore leads to high power consumption [12]. Frequency and time–frequency features also produce high-dimensional feature vectors, while time domain features are usually computationally efficient and suitable for edge feature extraction. Nine simple time domain parameters extracted at the edge have recently been investigated by [13] for wireless machine fault diagnosis. However, for SHM applications, the time domain features are sometimes less sensitive or robust to environmental and operational effects as compared to their frequency and time–frequency counterparts. Obtaining features that are sensitive to damage and can be implemented at the edge brings a new dimension to research and analysis in SHM.

It is clear that directly implementable solutions at the edge with demonstrated benchmarks are needed for the SHM sector to make a paradigm shift to an IoT-based future, and to accommodate the lifetime monitoring demands, combined with new detection algorithms. To address this overarching need, this paper developed a low-cost, low-power SHM prototype using off-the-shelf components within an open-source IoT framework. The prototype is deployed on an experimental cantilever structure excited by an electromagnetic shaker with damage being introduced into the system through the loosening of bolts at the fixed end of the cantilever. A detailed analysis of the raw vibration data gathered during the experiment is provided as a baseline for the features on the edge sensor node. This work demonstrates how a continuous structural monitoring and damage detection strategy can be achieved in an LPWAN using edge-computed features.

## 2. Materials and Methods

This section describes the system design of the Low-Power SHM setup and the analytical model of the experimental cantilever structure.

### 2.1. System Design and a Prototype of Low Power SHM

The low-power wireless sensor system for SHM in this paper consists of an edge device and a gateway. The edge device consists of sensors, a microcontroller and a wireless LoRa transceiver. The gateway, situated within the range of the edge device, consists of a LoRa gateway receiver, a microprocessor and a Wi-Fi or cellular module to communicate data to a cloud platform.

#### 2.1.1. Edge Device

**Figure 1:**
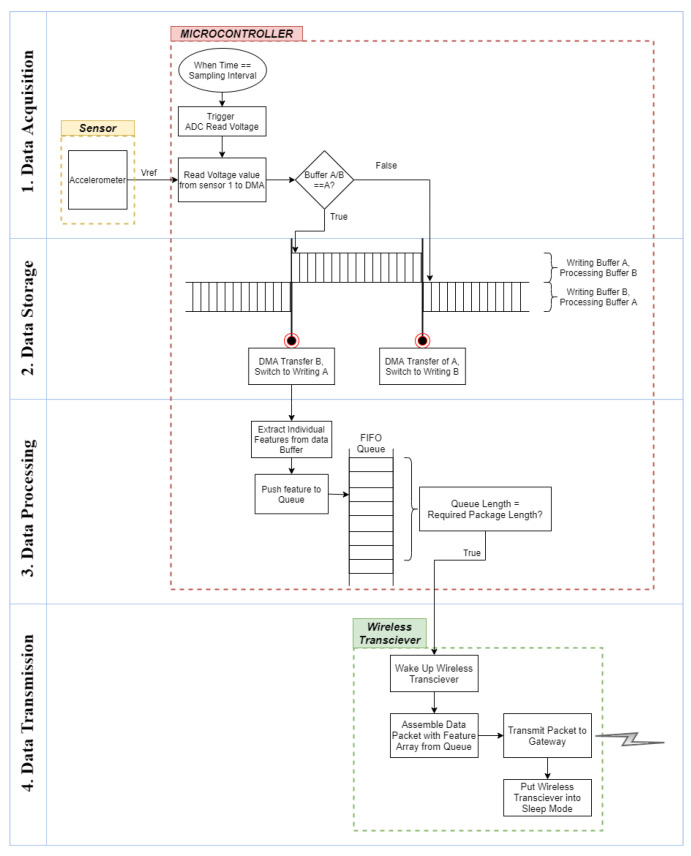
Edge device data flow.

The edge device continuously records data from three accelerometers. Due to the high sampling rate of the accelerometers and the transmission limits of low-power wireless communication protocols, the data are first locally stored and processed before features are extracted and wirelessly transmitted in a batch via LoRa. The data, following the acquisition, are stored, processed and subsequently transmitted as shown in Figure 1.

For *data acquisition*, a data-ready interrupt is used to sample voltage from the accelerometer at a set interval corresponding to the desired sampling frequency. Using the inbuilt timer in the microcontroller, the Analog to Digital Converter (ADC) is triggered at the pre-set sampling interval and the ADC reads the reference voltage (*V_ref_*) value for the analog pin connected to the sensor. An accelerometer, as a slave device, only records the voltage on command from the master device. Depending on the number of ADCs in the microcontroller, for multiple analog inputs, such as multiple sensors or multiple axis, the ADC must switch over each analog pin during the sampling interval. For *data storage*, a double buffering technique is used to simultaneously sample incoming data and carry out signal processing and data transmission on already-recorded data. The Direct Memory Access (DMA) controller is used to read the datapoint and store it in a buffer. Once the buffer is full, the data array is transferred for processing while a second empty buffer is filled. For *data processing*, signal processing and feature extraction are carried out on the complete buffer and calculated features are sent to a First In First Out (FIFO) queue. Once sufficient values are stored in the queue, they are assembled into a data packet and encoded as ASCII data for the RF payload. Finally, for *data transmission*, the LoRa transceiver module is switched on from sleep mode and used to transmit the assembled data packet to the gateway, returning back to a sleep state once the transmission has been successful.

#### 2.1.2. LoRa Data Transmission

LoRa is a noncellular radiofrequency carrier signal, which encodes information using a chirp spread spectrum (CSS) modulation scheme, enabling data communication over a long range (1–4 km in dense and up to 45 km in low density areas) with low power and minimum throughput. It is also the hardware that supports the modulation technology, including the LoRa chips and gateways, and is the physical layer in a Low-Power Wide-Area Network (LPWAN) system. A private LoRa network can be deployed for single applications leading to the advantage of larger message capacity compared to a public LoRaWAN gateway due to exclusive bandwidth, complete control over the end-to-end data transmission and the ability for bidirectional command and control functionality to the gateway and edge devices.

LoRaWAN is the media access control (MAC)-layer protocol communication, which is built on LoRa modulation technology and hardware. The LoRaWAN network architecture is laid out in a star-of-stars topology with a central gateway and multiple edge nodes in the network. It is best suited for public wide-area networks (WAN) as all the channels are tuned to the same frequencies, and its primary advantage is that only the edge sensor needs to be deployed in a monitoring application. However, they are limited by fair usage and access policies. The installation of a private gateway is necessary when a LoRaWAN network is not available in the region of deployment.

**Figure 2:**
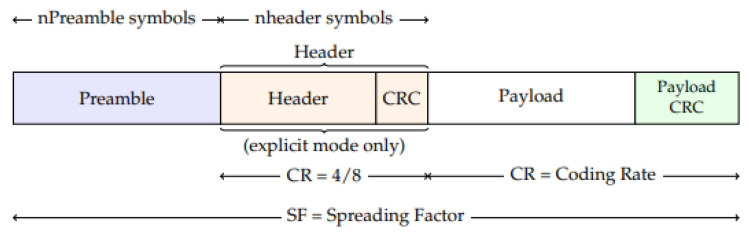
LoRa frame format (adapted from [14]).

LoRa (Figure 2) is linked to spread spectrum modulation where data can be spread in both frequency and time to increase the robustness and range of transmission by increasing the receiver’s sensitivity. The range and throughput of data transmission depend on the physical bandwidth for radiofrequency modulation (BW), coding rate (CR) and spreading factor (SF).

Larger bandwidths allow for a higher effective data rate, which reduces the transmission time but also reduces the sensitivity. The CR is for Forward Error Correction (FEC), which is combined with the spread spectrum technique to further increase the receiver sensitivity and correction. The SF affects the rate of data transmission, while the LoRa supports multiple spreading factors (between 7–12) to decide the tradeoff between the range and data rate. A lower SF results in a higher data transmission rate but also a lower range of transmission due to the reduced immunity to interference [15]. The data rate ranges from 300 bps to 37.5 kbps depending on the spreading factor and channel bandwidth [16] as

(1) $$f\left(PL\right)=\max\left(\left(\frac{PL+Header+CRC-4SF-20H}{4\left(SF-2DE\right)}\right)CR,0\right)$$

An uplink LoRa packet (Figure 2) consists of a set of preamble symbols, an optional header, a variable-length payload field and an optional cyclic redundancy check (CRC) field. PL represents the number of payload bytes, and the header is composed of preloaded information. The LoRa frame format can be either implicit or explicit, where the explicit packet includes a short header containing information about the bytes, CRC and coding rate used in the frame.

The data rate (DR) is defined by SF and BW, so the maximum packet size roughly depends on the distance to the nearest gateway. As LoRa operates in the unlicensed scientific bands, the DR is also limited by the specification for each region. For the European 863–870 MHz band, the maximum application packet size varies from 51 bytes for slower DR to 222 bytes for faster rates. The Header is composed of preloaded information, and DE indicates the absence (0) or presence (1) of the header in the packet.

#### 2.1.3. LoRa Gateway

The gateway or base station has a much higher communication capability, processing power and memory than the wireless senor node and is situated in a location within the range of the edge devices where power supply is not an issue. The gateway receives and parses the LoRa data packets and transmits them to a cloud data-management platform. For LoRa, the gateway can be private (user implemented) or public (LoRaWAN). In this paper, a private LoRa gateway setup is implemented, which consists of a microprocessor, a wireless LoRa receiver and an internet connection via a cellular or WiFi module. The LoRa gateway transceiver can receive data from multiple edge devices in a one-to-one star topology. The gateway is not a low-power setup and needs to be connected to a mains power source. It is in a constant listening state for incoming data packets from the edge nodes. These data blocks are parsed and transmitted via Wifi or 4G to a cloud IoT management dashboard. Data can then be stored, analysed and displayed on a dashboard on any IoT management platform.

### 2.2. Analytical Model of Experimental Setup

A replicable damage scenario is considered in this paper for the purpose of demonstrating SHM in an edge framework. Relaxation of the boundary condition of a cantilever structure, excited via an electrodynamic shaker, is considered as the damage scenario in this paper.

An analytical model is considered first to create the context and the relevance of the experiments. Relaxation of the boundary has seen several applications, including wind turbines [17], bridge scour [18] and damages related to other natural hazards [19,20]. The non-dimensionalisation and similitude relationships of such models are also established in the literature [17,21,22,23].

However, even within this idealised scenario, there are multiple analytical models to align with the variability that can exist within an experiment. The simplest reference case is a cantilever beam with a fixed end at the connection to the frame. This also helps with interpreting the data following the experiments. The second case is a two-span cantilever where the shaker probe acts as an internal connection. The third case is where the bolts are loosened, and the cantilever connection can be modelled using translational and rotational springs. The following sub-sections present these idealised models. 

#### 2.2.1. Cantilever Beam with Fixed End

The first natural frequency of a cantilever beam with fixed supports and an end mass is simply

(2) $$\omega_{cantilever}=\sqrt{\frac{k}{m_{eq}}}$$

where $k=\frac{3EI}{L^{3}}$ and $m_{eq}$ is the equivalent mass equal to $\frac{33}{140}\mathrm{mL}+M$, where m is the mass per unit length of the beam, L is the length of the beam, EI is the flexural rigidity of the beam and M is the tip mass at the end of the beam. 

#### 2.2.2. Two Span Cantilever Beam with Fixed End and Internal Connection: Fixed–Fixed–Free Boundary

**Figure 3:**
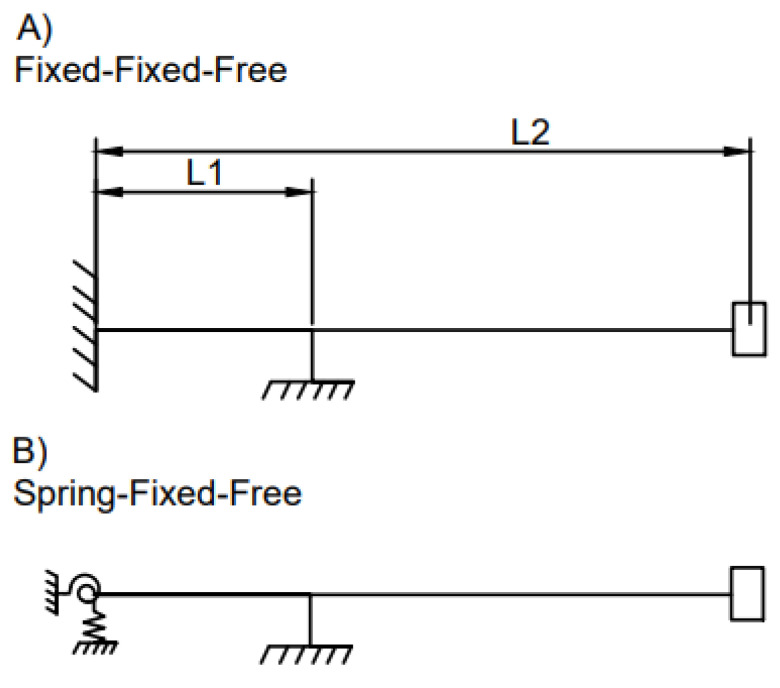
Two span continuous cantilever beams: (**A**) Fixed–fixed–free, (**B**) Spring–fixed–free.

The electromagnetic shaker is connected to the cantilever via a stinger, which is clamped to the beam so that vibro-impact nonlinearities are not induced. However, this creates an additional internal boundary condition on the beam, which makes the system a two-span continuous Euler–Bernoulli beam (Figure 3A), following the canonical equation

(3) $$\frac{\partial^{2}}{\partial x^{2}}\left(EI\left(x\right)\frac{\partial^{2}y\left(x,t\right)}{\partial x^{2}}\right)+\frac{\partial }{\partial x}\left(P\frac{\partial y\left(x,t\right)}{\partial x}\right)-\frac{\partial }{\partial x}\left(m\left(x\right)r^{2}\frac{\partial \ddot{y}\left(x,t\right)}{\partial x}\right)+m\left(x\right)\ddot{y}\left(x,t\right)=f\left(x,t\right)$$

where y(x,t) is the deformation of the beam with respect to its static deflected shape, f(x,t) is the time-varying load on the beam, x is the spatial coordinate along the length of the beam measured from the fixed end, t is time measured from the initiation of excitation, P is the constant axial force applied to the beam (which is made as close to 0 as possible), r is the radius of gyration of the beam and an overdot represents the derivative with respect to time. Appendix D provides details of the simplification of this equation considering boundary conditions.

#### 2.2.3. Two-Span Cantilever Beam with Loose Bolts and an Internal Connection: Spring–Fixed–Free Boundary

When bolts are loosened at the secured end of the cantilever, the connection is no longer a fixed connection as some vertical and rotational movement is expected to occur at the support. The secured end of the cantilever beam can therefore be described as a combination of a rotational and lateral spring (Figure 3B). This will affect the boundary conditions at the fixed end. Thus, at $x_{1}=0$, for loose-bolt spring connection

(4) $$EI\frac{\partial^{2}y_{1}\left(0,t\right)}{\partial x_{1}^{2}}-k_{r}\frac{\partial \left(0,t\right)}{\partial x_{1}}=0$$

(5) $$EI\frac{\partial^{3}y_{1}\left(0,t\right)}{\partial x_{1}^{3}}+P\frac{\partial y_{1}\left(0,t\right)}{\partial x_{1}}+k_{l}w\left(x_{1},t\right)-mr^{2}\frac{\partial^{2}\ddot{y_{1}}\left(0,t\right)}{\partial x_{1}^{2}}=0$$

where $k_{r}$ and $k_{l}$ are the rotational and lateral stiffness of the springs representing the loosened bolted cantilever connection, respectively.

### 2.3. Experimental Setup

**Figure 4:**
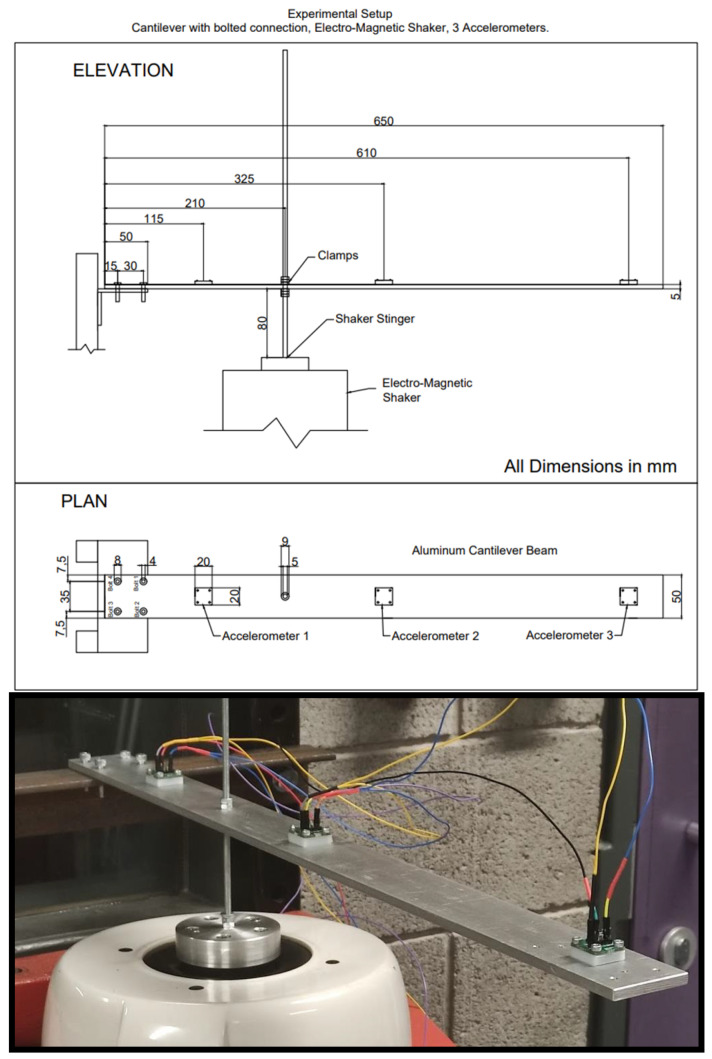
Experiment setup with shaker, cantilever and sensors connected to Arduino Due.

The experimental setup contains a cantilever beam secured by a bolted connection to a frame. The cantilever, a 650 mm × 50 mm × 5 mm aluminium beam secured to a frame by four bolts of 4 mm diameter, is excited by a sensor probe connected to an electrodynamic shaker at one-third distance along the beam. Three accelerometers of 8 g mass are connected to the cantilever on both sides of the probe and at the free end. Damage is introduced into the system through the loosening of bolts at the fixed end. Figure 4 presents the experimental setup.

**Figure 5:**
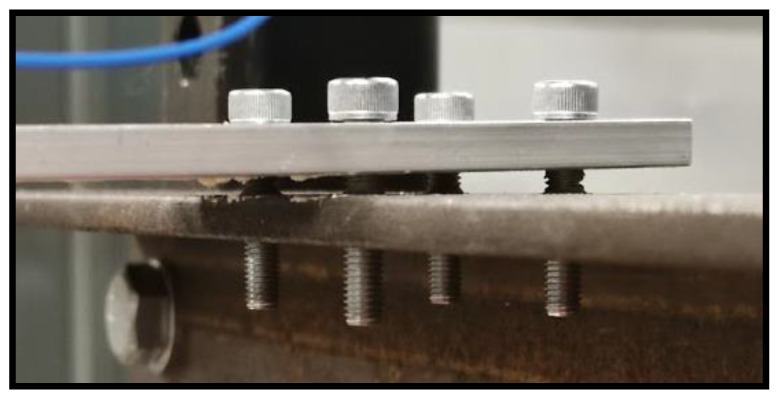
Loosened bolts at cantilever fixed end.

The natural frequency of the cantilever is analytically calculated as 11.06 Hz. With the attached stinger and the change in boundary conditions, the natural frequency is computed to be 20.398 Hz. These values correspond to numerical and experimental work on two-span beams in [24,25]. Increasing and decreasing swept sine, along with white noise excitation, are used to excite the beam for each bolt-loosening incident at the fixed end of the cantilever (Figure 5), in a frequency range of 5–200 Hz and for 5 s. Table A1 and Table A2 in Appendix A show the progression of bolt loosening for the two sets of damage experiments. 

Subsequently, continuously recorded experiments were also carried out for 2 min as the bolts were progressively loosened. While the fundamental physics of bolt loosening can be particularly complex [26], the gradual loosening and its relationship to SHM is well established, as we consider this deviation from the undamaged baseline as the feature of interest in this paper. Such experiments can also typically be created as rapid benchmarks [27]. 

**Figure 6:**
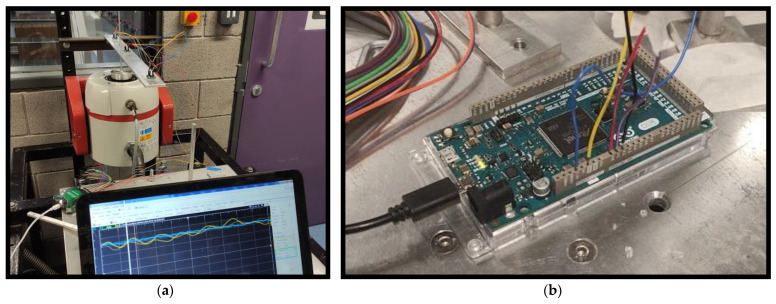

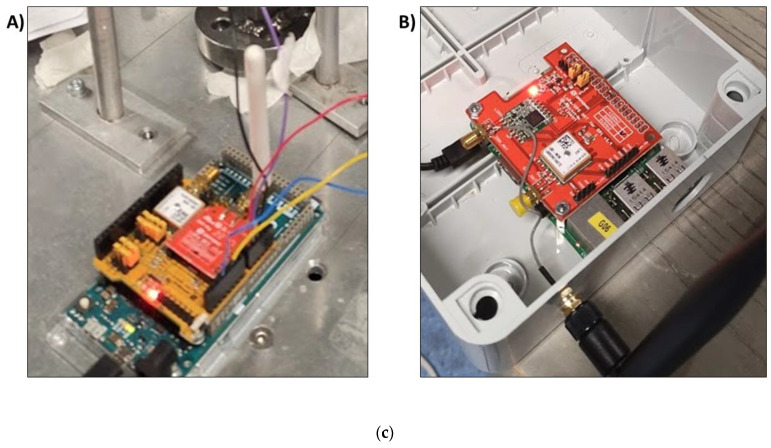
(**a**): Oscilloscope Realtime display from accelerometers; (**b**): Arduino connected via analog inputs to accelerometers; (**c**): LoRa transmission via (**A**) edge device, Arduino microcontroller with LoRa shield; (**B**) gateway, raspberry Pi microprocessor with LoRa receiver hat.

Data were collected in three different ways. First, an oscilloscope monitored each shaker input and recorded the raw acceleration data at 500 Hz. Secondly, each accelerometer was connected to the analog inputs of the Arduino Due used for the experiments, where the values are printed via the USB serial port to a local PC. This method uses the Arduino Due as an oscilloscope. Finally, the LoRa setup described was used to extract root mean square (RMS) features from the raw data and transmit them to a local PC via the LoRa gateway. Figure 6 summarizes the data collection methods.

### 2.4. SHM Low-Power IoT Framework

**Figure 7:**
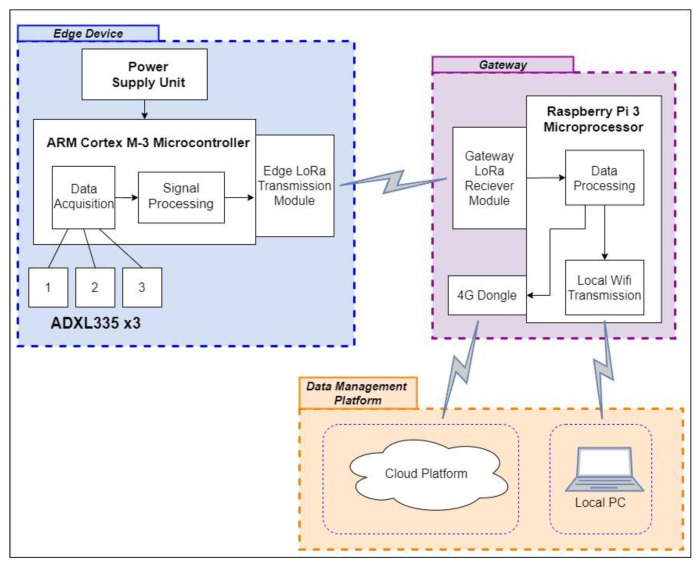
Low-power SHM framework.

Figure 7 shows the three sections in the IoT framework—the edge device, gateway and data management platform used in this paper. The LoRa transmission settings and gateway configuration in this application are based on the low-cost and low-power IoT framework developed in the H2020 EU WAZIUP project [28,29].

For the edge device setup, the Arduino Due microcontroller was used, which has a single built-in ADC and *V_ref_* = 3.3 V. The Arduino samples the data from the accelerometers at 500 Hz. The Arduino Due is a 32-bit CortexM3 ARM microcontroller with an 84 MHz clock. A shield is attached to the microcontroller for LoRa communications. A 3-axis, ±3 g, ADXL335 micro-electromechanical systems analog accelerometer is used for data measurement. The evaluation board is used for the prototype to easily connect the accelerometer to the pins of the microcontroller (Raspberry PI 3). The accelerometer has a 350 µA power consumption and a 0.5–1600 Hz measurement range on the X and Y axes, and a 0.5–550 Hz range on the Z axis, respectively. The ADXL335 has a ratiometric output voltage, *V_s_* of −0.3–3.6 V and for a 10-bit ADC resolution (2^10^), the acceleration value in g is:

(6) $$acceleration\;\left(g\right)=\frac{V\times V_{s}}{2^{10}}$$

However, the accelerometer has an offset bias of $V_{s}/2$. For the 3.3 V supply from the Arduino, the sensitivity of the sensor is 270–330 mV/g and the acceleration is

(7) $$acceleration\;\left(g\right)=\frac{1}{sensitivity}\left(\frac{V\times V_{s}}{2^{10}}-\frac{V_{ref}}{2}\right)$$

This value must be determined for each sensor before the measurement is carried out. For the three accelerometers used in this experiment, the sensitivity values were 305 mV/g, 302 mV/g and 299 mV/g for accelerometers 1, 2 and 3, respectively.

Feature extraction at the edge is required due to limits on data packet sizes, and this paper considers the RMS value and the peak natural frequency as extracted features.

For long-term continuous monitoring, features need to be either calculated or averaged over time windows that can be transmitted in small packets over intervals of several minutes. For the EU863–870 unlicensed bands, the maximum available payload size per LoRa message is 222 bytes for a data rate of 4–7 (BW 125, CR 5, SF 12). To obtain enough samples, features are calculated over 1 s of recording. Using the above configurations and a maximum payload of 222 bytes, a payload of 128 bytes for the data packet is used. An 8-bit ASCII encoding is considered, and each feature is stored to flash memory (512 kB). Data are queued to be sent out at 3 min intervals at a later time. In a full-scale scenario, the interval over which the features are calculated would be much longer. 

The Arduino Due is a 3.3 V microcontroller and has an estimated power consumption of 100 mA. The 3 accelerometers are powered by the Arduino, and each has a current draw of 0.35 mA. The LoRa shield has a current draw of 20 to 120 mA while transmitting (depending on the boosting for the maximising range) and 0.2 micro amps while in sleep mode. That is an estimated range of 0.4 W to 0.7 W while the LoRa device is transmitting and 0.3 W while the accelerometers are recording and the LoRa device is in sleep mode. 

Field implementation of this prototype would require the development of an Application-Specific Integrated Circuit (ASIC) with an application-specific code to replace the Arduino Due microcontroller, significantly reducing the power draw.

## 3. Results

As indicated in the previous section, bolts are first loosened in discrete stages in the first set of experiments, which is subsequently followed by a set of tests where continuous and progressive loosening is carried out while the shaker excites the system via swept sine and white noise (5–200 Hz), respectively.

### 3.1. Discontinuous Measurements

#### 3.1.1. White Noise Input

**Figure 8:**
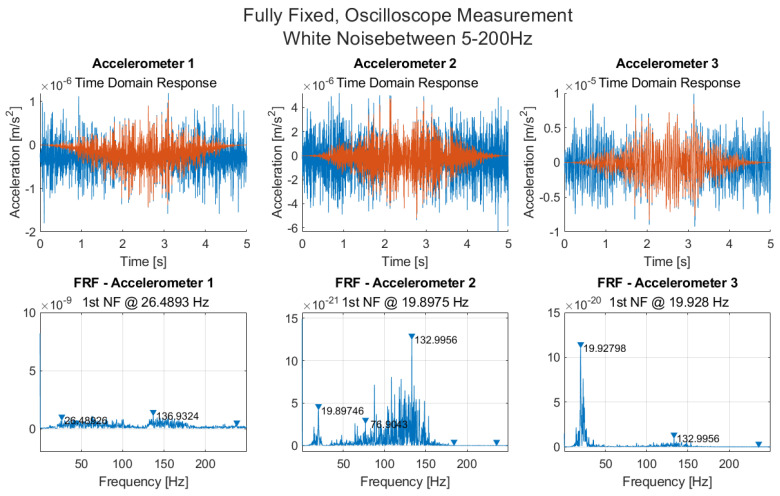
Time and frequency domain measurements, along with measured FRFs from accelerometers 1, 2 and 3 for white noise excitation using an oscilloscope.

The frequency response functions (FRF) are obtained using a discrete Fourier transform (DFT) from the raw data (Figure 8). A Hanning window is applied to the data for noise reduction from spectral leakage. The effect of the Hanning window applied to the 5 s time interval can also be distinguished here. 

**Figure A1:**
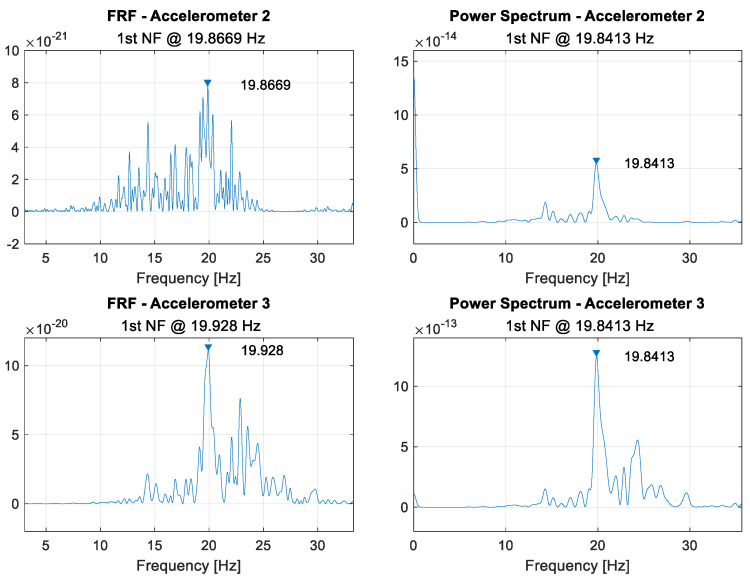
Close-up around first natural frequency, oscilloscope measurement white noise input, FRF and power spectrum plots, accelerometers 2 and 3.

For Accelerometer 1, there are no significant peaks, and for accelerometers 2 and 3, the first natural frequency is at 19.9 Hz. Figure A1 in Appendix B shows a close-up around the first natural frequency for the FRF and power spectrum plots for accelerometers 2 and 3. For accelerometer 3, the FRF and power spectrum plots show evidence of a double peak.

#### 3.1.2. Arduino Measurement

**Figure 9:**
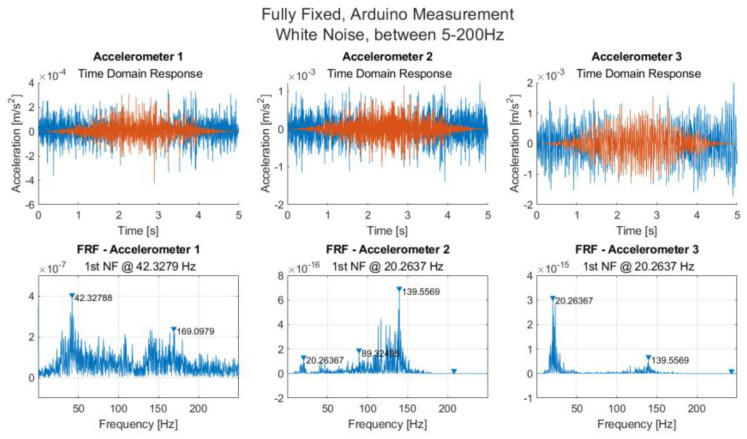
Time domain measurements along with measured FRFs from accelerometers 1, 2 and 3 for white noise excitation using an Arduino Due.

Figure 9 shows a plot of the time domain response, the frequency response function and a spectrum plot for each of the accelerometers 1, 2 and 3 recorded using Arduino.

**Figure A2:**
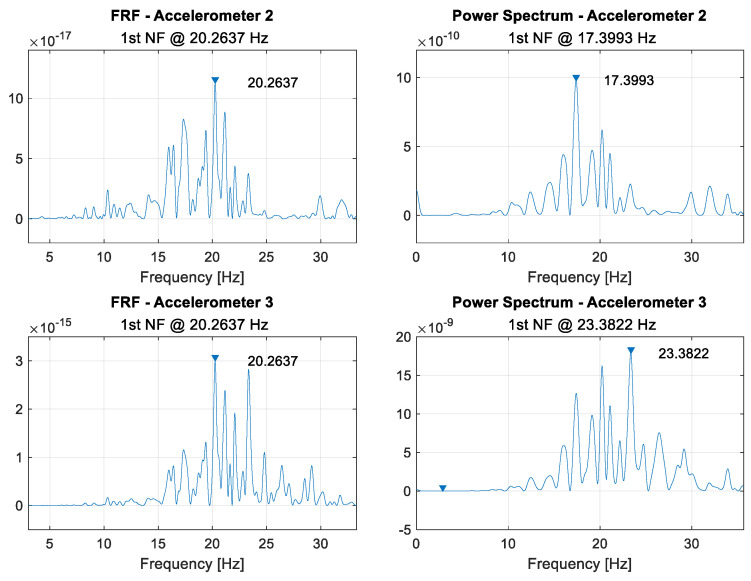
Close-up around first natural frequency, Arduino measurement white noise input, FRF and power spectrum plots, accelerometers 2 and 3.

Here, the first natural frequency is 20.2 Hz, which is 0.3 Hz higher than that observed with the oscilloscope, and the evidence of double peaks is more obvious when a close-up is considered (Figure A2, Appendix B). For accelerometer 2, the power spectrum plot shows a peak at 17.3 Hz rather than 20.2 Hz, and for Accelerometer 3, a maximum peak of 23.3 Hz is detected for the power spectrum plot. For the FRF of Accelerometer 3, the second peak, although lower, is almost as high as the natural frequency at 20.2 Hz. Similar plots are shown for each of the bolt-loosening scenarios in Appendix B. 

#### 3.1.3. Three-Dimensional Plots of Each Bolt-Loosening Scenario

**Figure 10:**
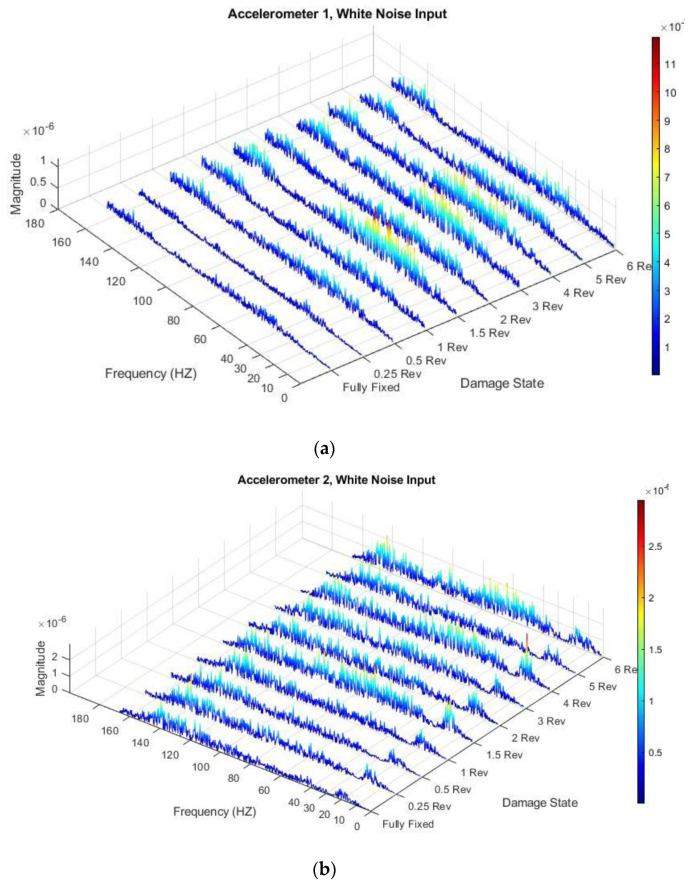

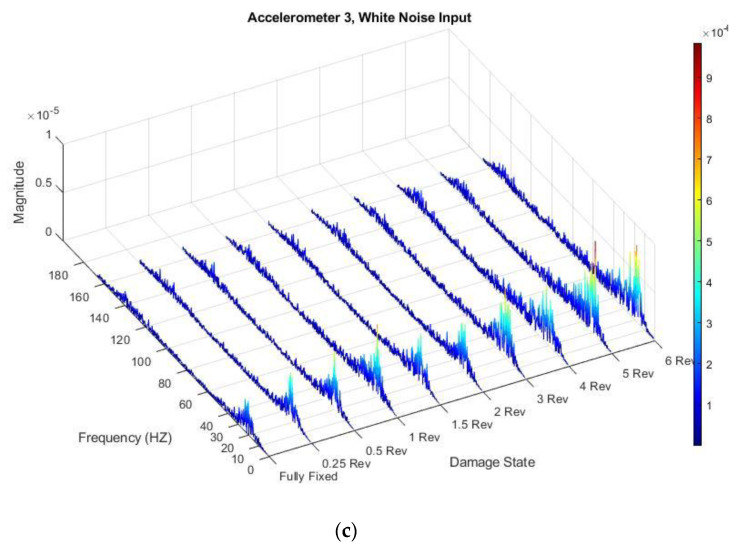
FRF of Arduino measurement for each bolt-loosening scenario (Damage State) with a white noise input for (**a**) Accelerometer 1; (**b**) Accelerometer 2 and (**c**) Accelerometer 3.

The frequency response from each of the bolt-loosening scenarios are shown for the Arduino measurement for accelerometers 1, 2 and 3 with a white noise input in Figure 10.

The magnitude of the frequency response increases significantly as the bolts are loosened. Although there is no clear peak natural frequency for Accelerometer 1, the highest region for the fully fixed case is centred around 40–45 Hz. This region shifts upwards as the bolts are loosened and becomes more distributed across a wider range of frequencies. The magnitude of the second peak region also increases with each bolt-loosening scenario relative to the fully fixed case. For Accelerometer 2, the magnitude of the frequency response increases significantly with the bolt loosening relative to the fully fixed scenario. From a loosening of 1.5 revolutions, a third peak appears to form around 70 Hz, particularly obvious for revolution 6 where the first natural frequency decreased compared to revolution 4. The first natural frequency is much more evident here than in the previous two figures as this accelerometer is located at the tip of the cantilever. There is a dispersion of the width of the frequency response and an increase in the magnitude of the response with each bolt-loosening scenario at the first natural frequency. Evidence of a double peak also becomes particularly clear from revolution 3 to bolt revolution 6.

**Figure 11:**
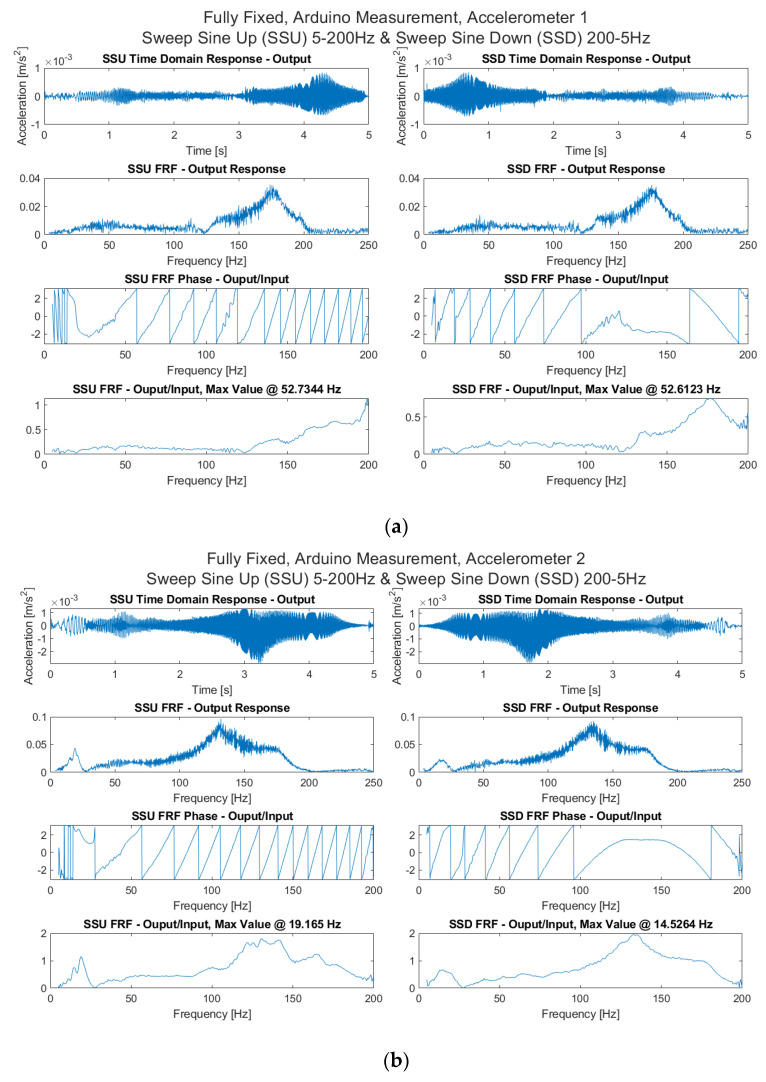

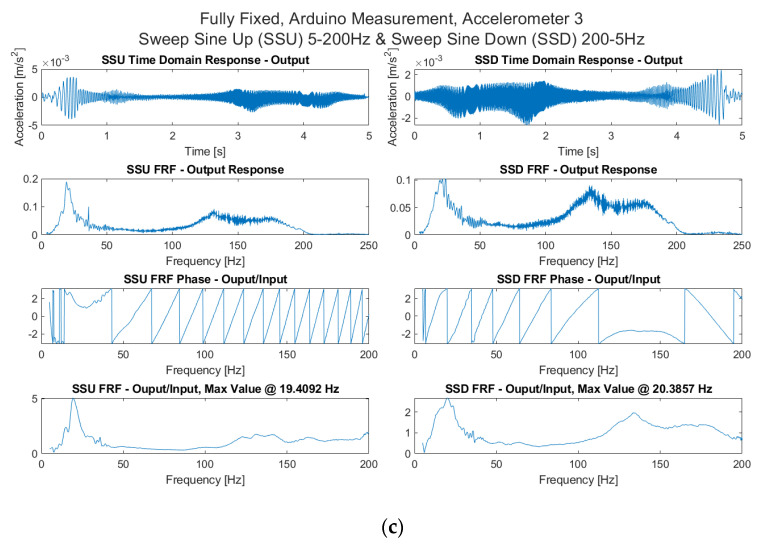
Time and frequency domain responses and FRF estimates from swept sine responses using Arduino measurement for all accelerometers: Fully fixed scenario for (**a**) Accelerometer 1; (**b**) Accelerometer 2 and (**c**) Accelerometer 3.

The FRFs for swept sine inputs (5–200 Hz, increasing and decreasing) are presented in Figure 11 for the accelerometers. Clear peaks for accelerometer 2 at 19.1 Hz for the upward sweep and 19.4 Hz and 20.3 Hz for accelerometer 3 for white noise inputs are obtained. It is important to note the variability of measurement obtained from an edge monitoring setup in this regard. It highlights the fundamental SHM challenges [30] of assessing full-scale structures, but also confirms the ability of such a setup to perform similar to what is possible in a traditional wired and wireless setup. 

**Figure 12:**
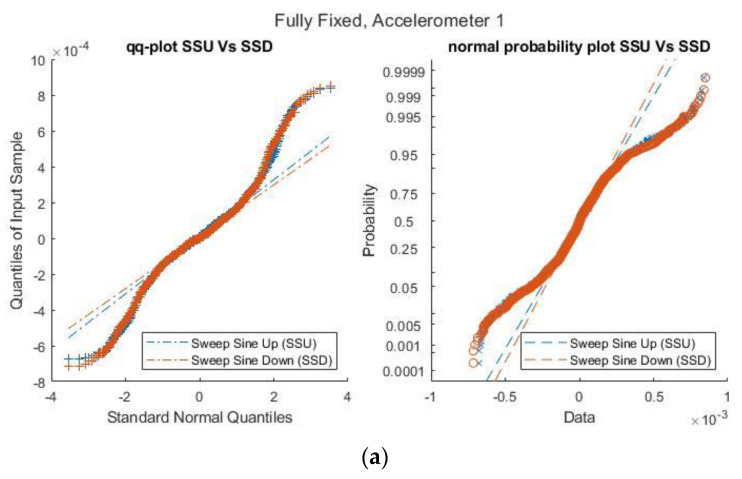

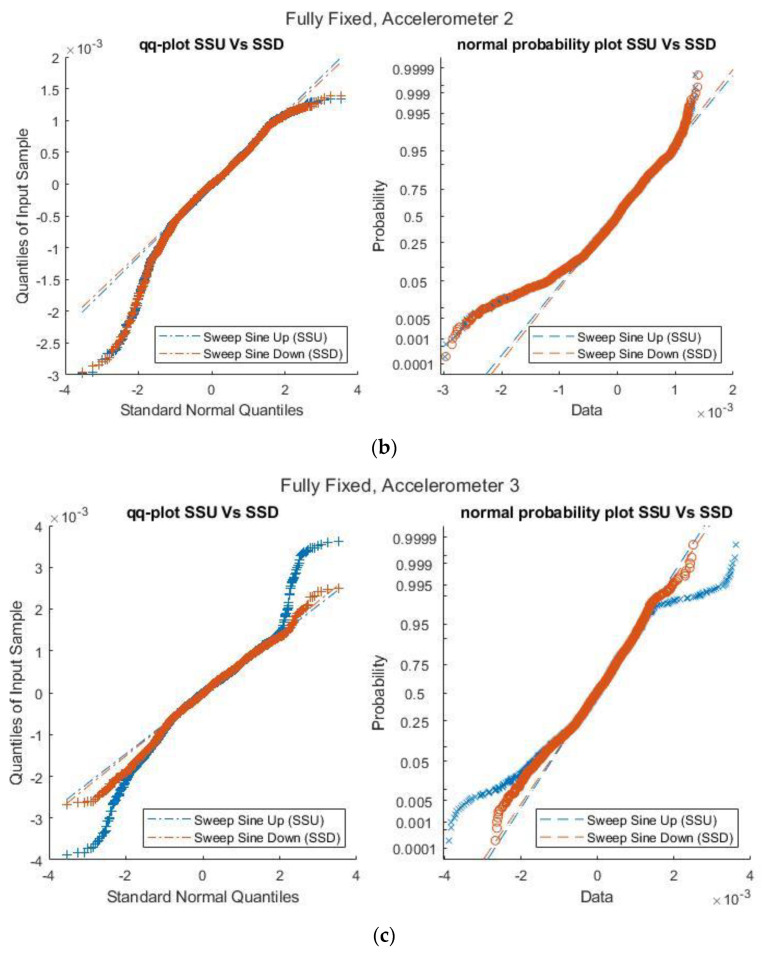
Q–Q and normal probability plots for SSU vs. SSD for accelerometers for (**a**) Accelerometer 1; (**b**) Accelerometer 2 and (**c**) Accelerometer 3.

Next, quantile–quantile plots and normal probability plots were created for the swept sine scenarios to investigate the impact of changes in the boundary condition brought about by the loosening of bolts for the accelerometers (Figure 12). There is a direct correlation between the SSU and SSF for accelerometers 1 and 2 and accelerometer 3, and this correlation only deviates outside two normal quantiles in the Q–Q plot and the tails of the distribution beyond 0.05 in the normal probability plot. This is also representative of the ability to use established statistical markers for monitoring features of interest [31] in an edge setup. Previous research has also indicated that such markers can accommodate traditional, as well as new [32], types of sensors.

#### 3.1.4. Continuous Measurement of Bolt Loosening

**Figure 13:**
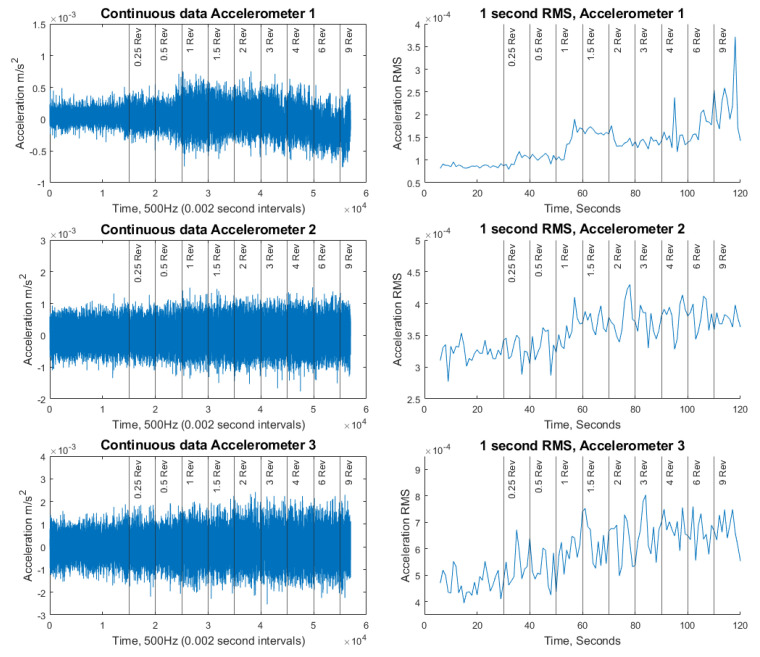
Raw accelerometer data and 1 s RMS for continuous recording of progressive bolt loosening for all accelerometers.

For the second set of experiments, where damage is introduced through the progressive loosening of bolts while the cantilever is being excited by the shaker, the Edge SHM prototype demonstrates the accelerometer data, along with 1-s RMS values (Figure 13). The revolution values shown in the plots are the accumulative revolution of loosening. 

## 4. Discussion

Some of the core challenges for SHM, identified more than a decade ago, are still relevant [33]. However, with a proliferation of sensors and the rise of IoT in most sectors [34], there is a definitive need for demonstrative and repeatable benchmarks for SHM, especially around edge computing, which this paper addresses. It impacts the value of information of [35,36,37] for existing and future sensors, as well as their ranges of demands. The potential use of IoT for SHM has been discussed previously [38], and despite architecture being discussed [39,40,41,42], there are only a handful of small examples [43] and no benchmark. To the best of the authors’ knowledge, this is the first demonstration with a clear architecture, design, repeatable benchmark and implementation of SHM in an edge-computing format, and this is expected to open up significant figure study in future. The presented method can accommodate most markers of features of interest and thus, there are significant future opportunities to adapt this approach to sector-specific requirements. The deviation of the quantile–quantile plots as a function of bolt loosening is indicative of this, especially in the context of output-only markers [44], and especially in a statistical quality control context [45].

The demonstrated edge SHM can be implemented in a low-cost, low-power format using off-the-shelf components and within an open-source IoT framework. The work demonstrates the constraints that a low-power remote-monitoring system places on data-driven SHM. Due to the low data-transmission rate available combined with the high sampling frequency of accelerometers, this work demonstrates the need for univariate features that can be extracted at the edge. The necessity for feature extraction at the edge is demonstrated due to the constraints of the low-power communication data rates. Open-source or commercial kits for remote SHM are not yet available, and one of the prohibitive challenges for researchers in gathering data and testing algorithms on a low-power system is the requirement of developing custom hardware solutions. An SHM prototype for this requires the design and development of a specialised hardware system. Some of the numerous parameters that need to be considered when implementing a low-power wireless monitoring system are identified through this work. These parameters include:

- Maximum size of each payload in the data packet for an application.
- Transmission interval for data packets.
- Whether to implement a sleep interval between recordings.
- Features to extract.
- Interval over which each feature is extracted.
- Averaging of features.

One of the limiting factors in this prototype on the number of acceleration channels that can be sampled in parallel using analog accelerometers is the number of analog-to-digital converters in the microcontroller. The Arduino Due used in this work has a single 12-bit resolution analog-to-digital converter. It is recommended for future developments that a digital mems accelerometer such as the ADXL345 with an inbuilt 16-bit resolution analog-to-digital converter be used instead. This digital accelerometer also has a lower power consumption.

**Figure A3:**
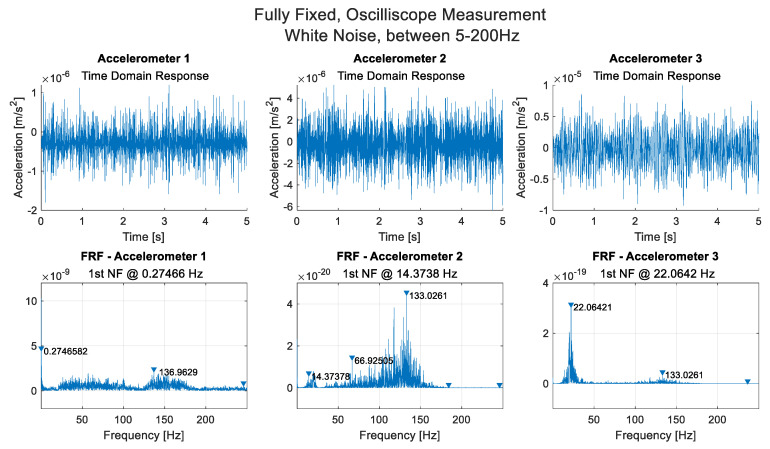
Oscilloscope measurement: Fully fixed boundary.

**Figure A4:**
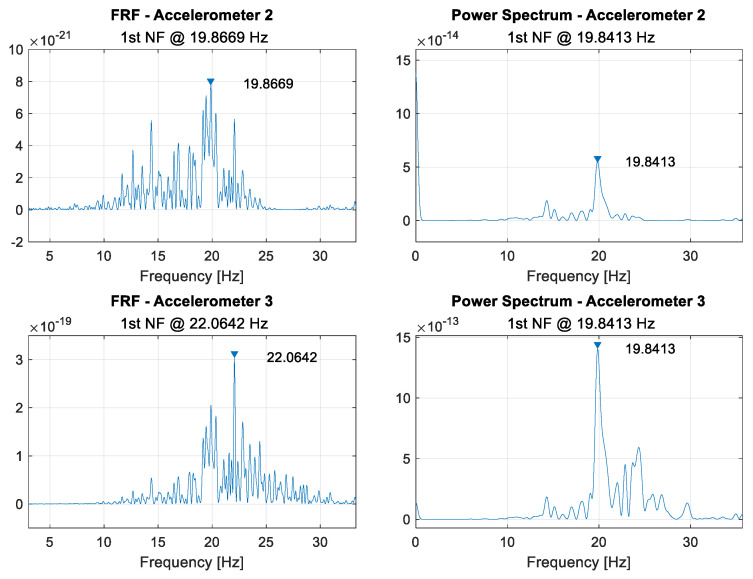
A close-up of FRF and the power spectrum plot. The peaks are at 22.06 Hz and 19.89 Hz from FRF and 23.9 Hz and 19.7 Hz for power spectrum, respectively.

**Figure A5:**
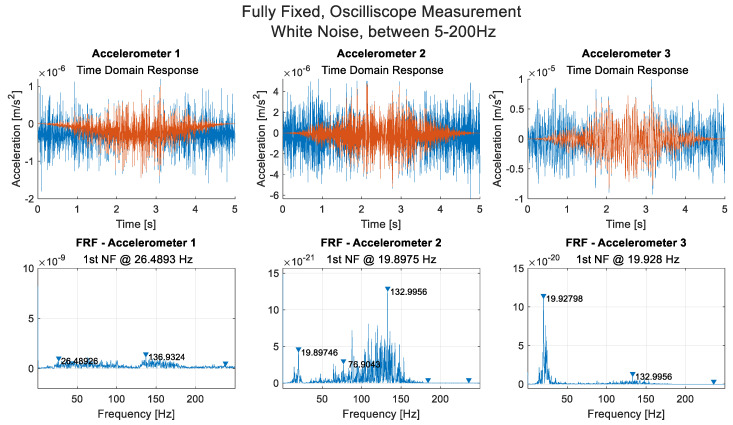
Oscilloscope measurement with Hanning window: Fully fixed boundary condition.

**Figure A6:**
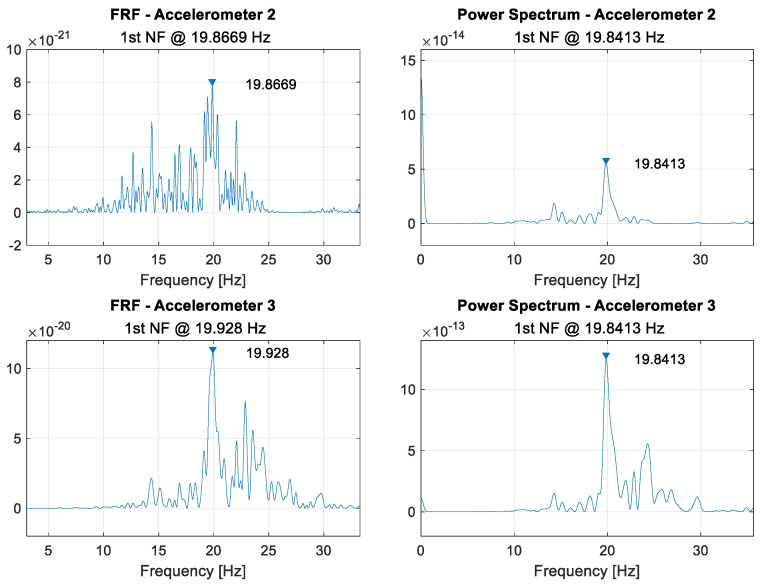
A close-up of FRF and the power spectrum plot. The peaks are at 19.928 Hz and 22.88 Hz for the FRF and 19.8 Hz and 24.23 Hz for the power spectrum, respectively.

**Figure A7:**
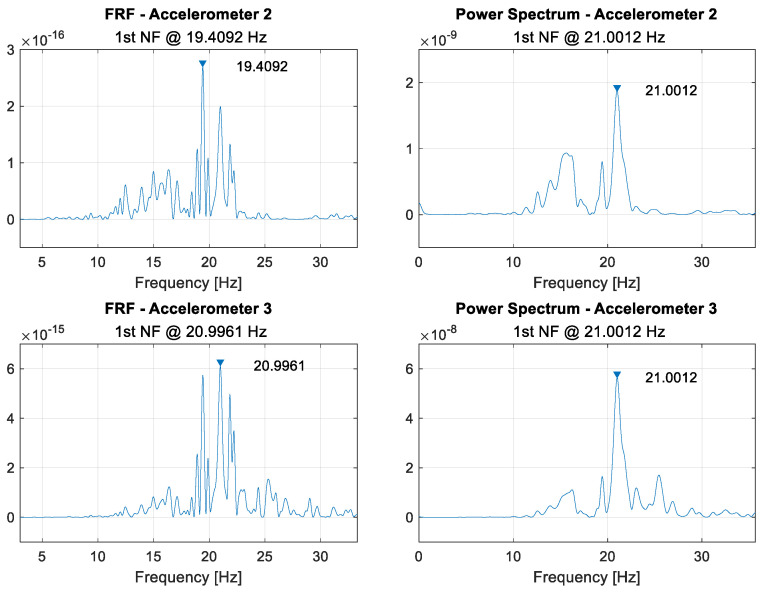
Arduino measurement: 0.25 revolution bolt release.

**Figure A8:**
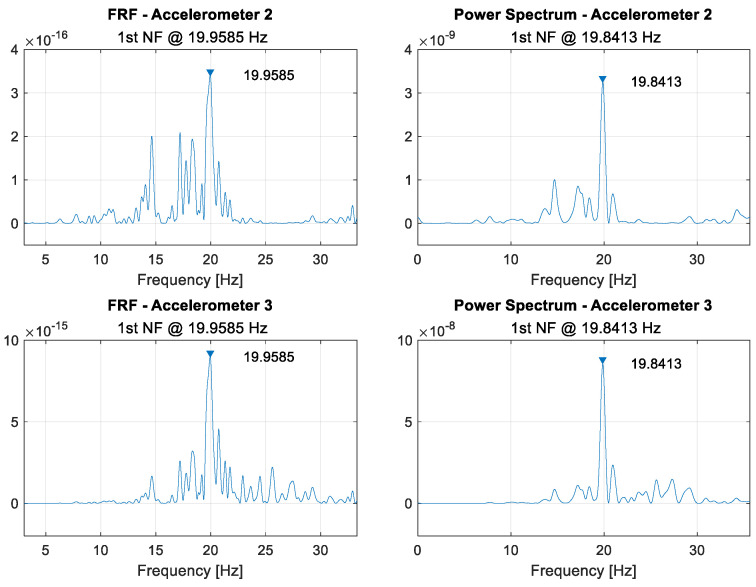
Arduino measurement: 0.5 revolution bolt release.

**Figure A9:**
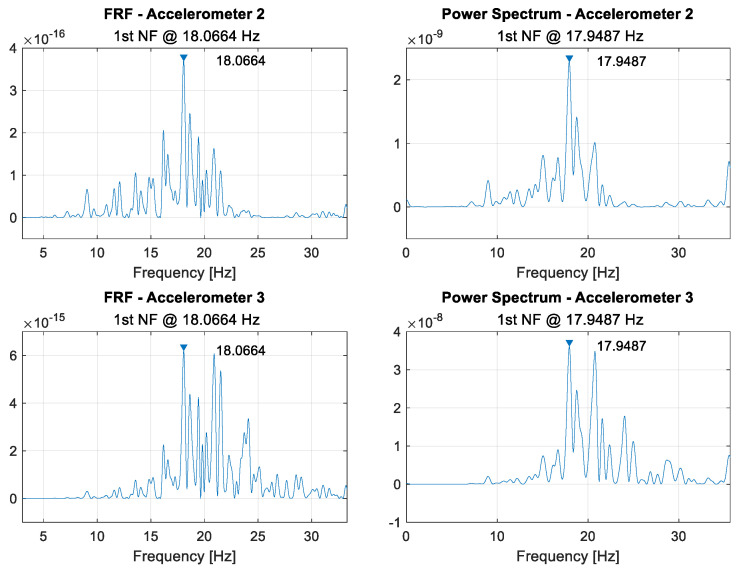
Arduino measurement: 1.0 revolution bolt release.

**Figure A10:**
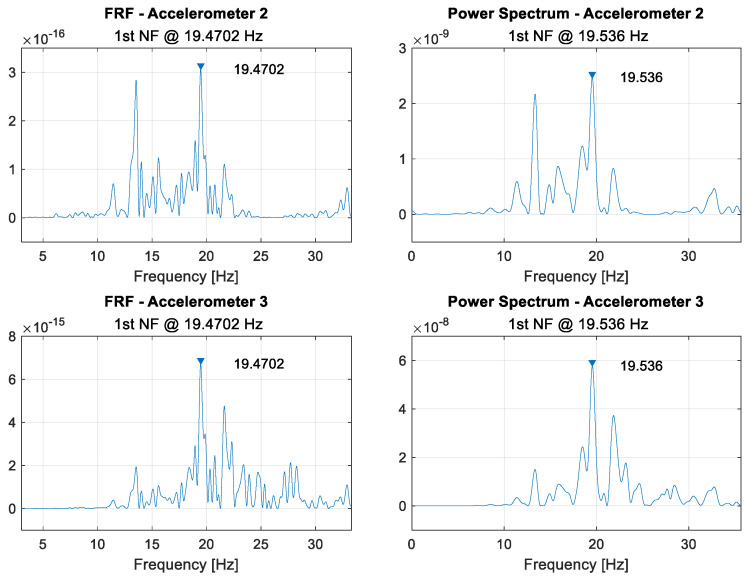
Arduino measurement: 1.5 revolution bolt release.

**Figure A11:**
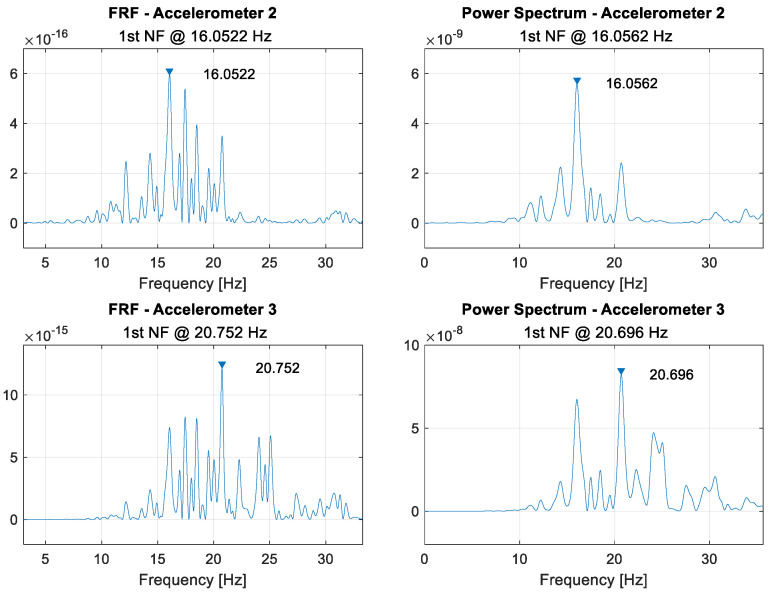
Arduino measurement: 2.0 revolution bolt release.

**Figure A12:**
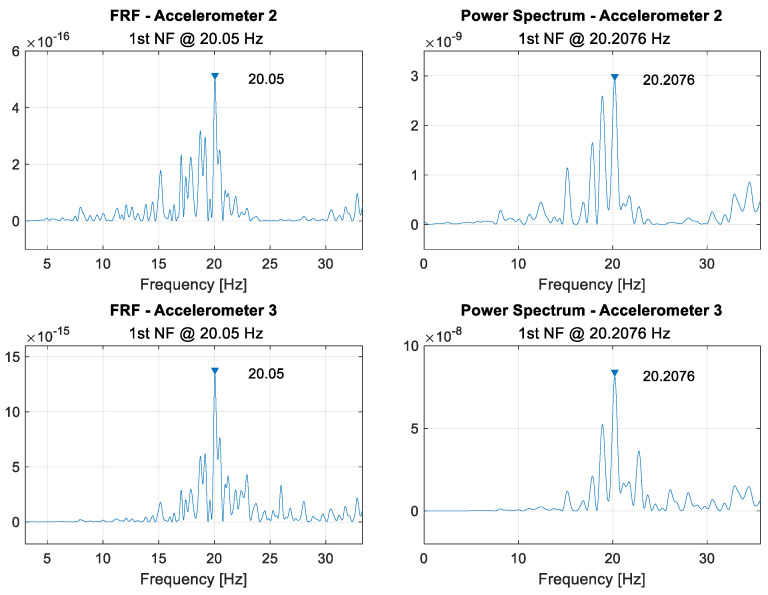
Arduino measurement: 3.0 revolution bolt release.

**Figure A13:**
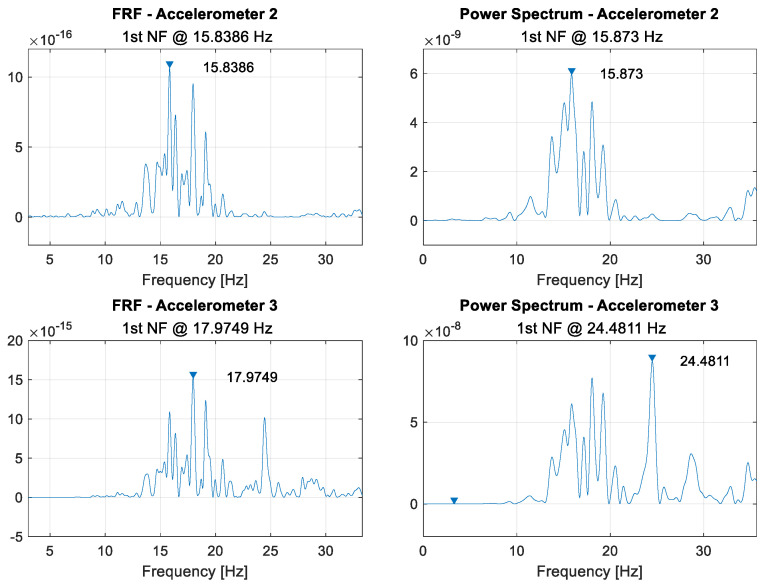
Arduino measurement: 4.0 revolution bolt release.

**Figure A14:**
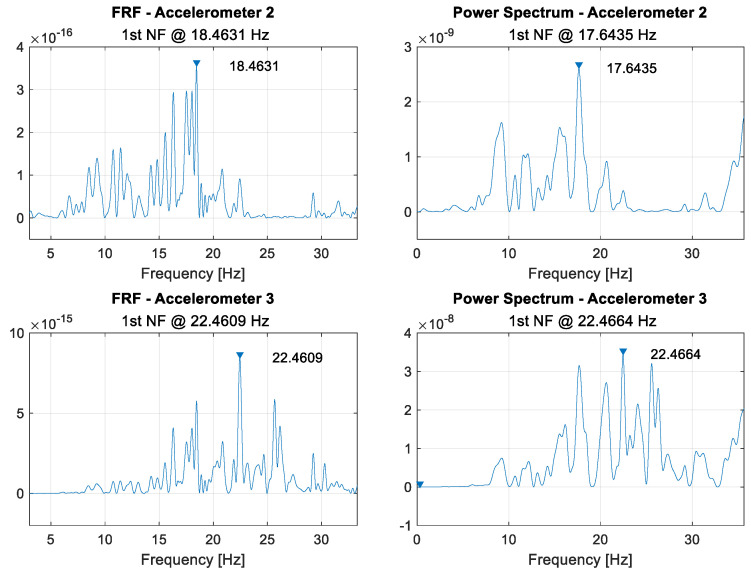
Arduino measurement: 5.0 revolution bolt release.

**Figure A15:**
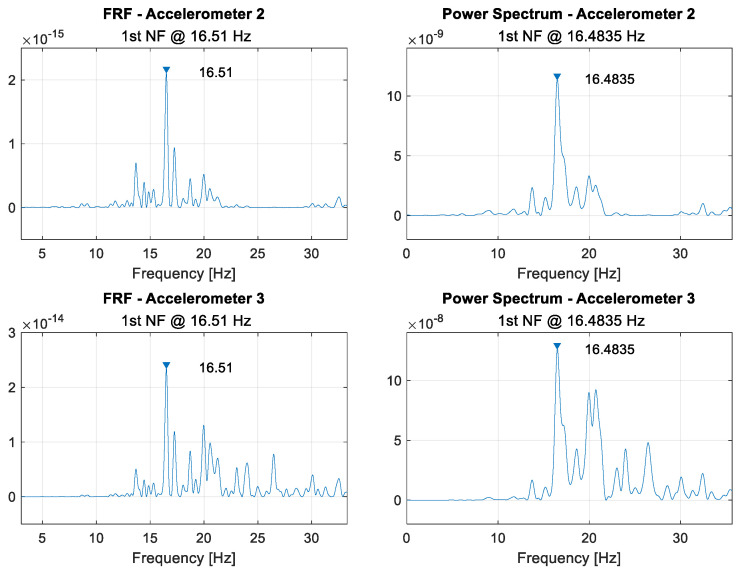
Arduino measurement: 6.0 revolution bolt release.

**Figure A16:**
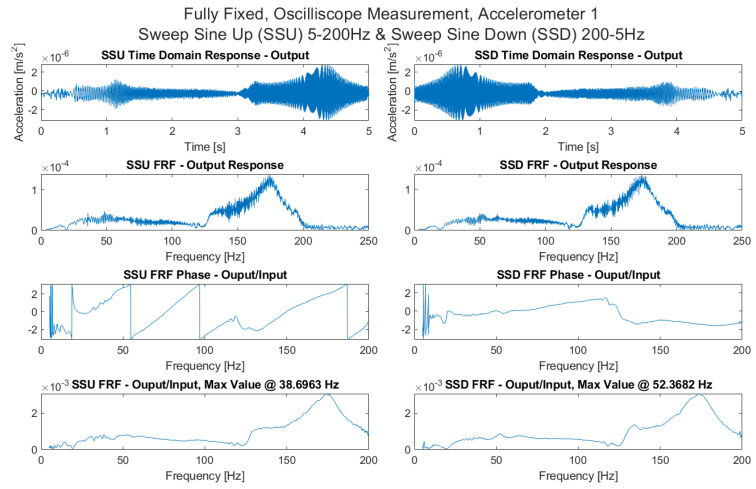
Swept sine inputs: Oscilloscope measurements: Accelerometer 1.

**Figure A17:**
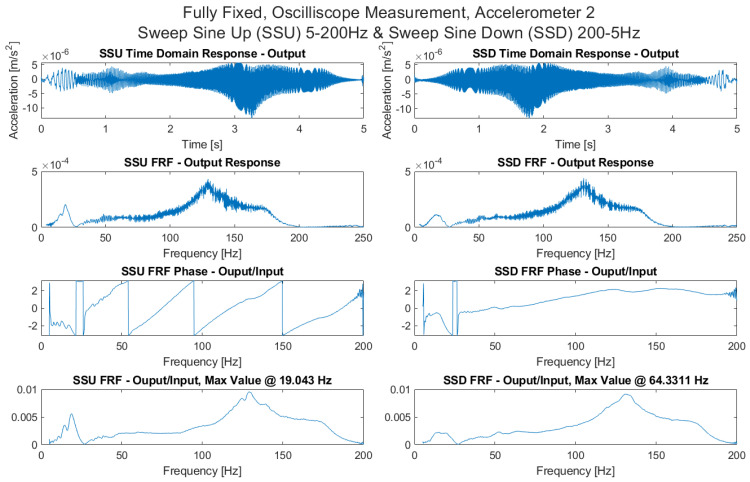
Swept sine inputs: Oscilloscope measurements: Accelerometer 2.

A baseline is still required for the features on the edge sensor node, but in the future, it may be possible to carry out some detections with multiple sensors, especially for anomalies, in order to not require an undamaged baseline [46]. The presence of double peaks is generally unavoidable in most experiments [47] and thus it is better to identify them. Such peaks are typically a result of the presence of coupling between the fixed beam and the stinger, since it is often not possible to e establish perfect rigidity, despite avoiding vibro-impact [48] conditions. Despite the simplicity and ease of repeatability of the experiments, there are possibly minor nonlinearities introduced into the system as the bolts are initially loosened and there can still be occasional and minor impacts between the beam and the frame and top of the bolts. As the bolts are further loosened, this would be reduced, as the bolts would be loose enough and the beam stiff enough to ensure no possibility of impact with the top of the bolt or the frame. There would still, however, be some noise effect due to the friction between the cantilever and the bolt. A set of additional results supporting the observations in this paper, and for the purpose of appreciating the consistency and repeatability of the study, is presented in Appendix C in Figure A3, Figure A4, Figure A5, Figure A6, Figure A7, Figure A8, Figure A9, Figure A10, Figure A11, Figure A12, Figure A13, Figure A14, Figure A15, Figure A16 and Figure A17.

The excitation profiles of swept sine and white noise are, on the other hand, typical of what is provided in industrial applications [23,49]. Consequently, experiments of a similar nature can be used at least phenomenologically to establish whether an edge SHM solution can be viable for the monitoring demands of a sector. The distance of certain renewable energy devices, like offshore wind [50], can provide an opportunity in this regard.

While extensive control on such experiments will improve the calibration of the results, there will always remain some uncertainties of these implementations, and there is a requirement to carry out further comparative studies in the future for various deployments of edge SHM and establish the uncertainty bounds for detection and the demands for such detection for a range of application sectors or use cases. On the other hand, the clear trend of the markers with bolt loosening indicates that a continuous structural monitoring and damage detection strategy can be achieved in an LPWAN using edge-computed features.

## 5. Conclusions

This paper presents a bespoke edge SHM framework and presents a benchmark study around it. The work creates a replicable evidence base for researchers around the topic of decentralized computing in SHM and can link with any feature analytics, along with single- and multi-channel markers of features. The work emphasizes the paradigm shift in SHM via low-power computing while highlighting practical challenges in managing data communication, high sampling rates and the necessity of carrying features at the location of a structure as much as possible. The method presented is scalable and can be applied to a wide range of sectors, both traditional and bourgeoning. The study will be relevant for industrial applications, as well as linking new numerical, statistical or learning algorithms to the edge framework, precipitating a new tranche of research activity. Sensing our built infrastructure and making sense of it can forge a new direction in edge solutions.

## Data Availability

Data for the experiments are available from the authors on request.

## Appendix A

**Table A1:** Experimental bolt-loosening progression experiment (discrete).

Experiment	Bolt Loosening: Revolutions	Bolt Loosening, Total Revolutions	Distance between Frame and Top of Bolt (mm)
1	0 (Fully Fixed)	0 (Fully Fixed)	5 mm (beam depth)
2	0.25	0.25	5.2
3	0.25	0.5	5.4
4	0.5	1	5.75
5	0.5	1.5	6.07
6	0.5	2	6.48
7	1	3	7.2
8	1	4	7.73
9	1	5	9.3
10	1	6	9.73
11	2	8	11.1

**Table A2:** Experimental bolt-loosening progression experiment (continuous).

Experiment Number	Bolt Loosening, Revolutions	Time Total, Seconds	Time (per Loosened Stage) Seconds
1	Fully Fixed (0 revolutions)	0	30
2	0.25	30	10
3	0.25	40	10
4	0.5	50	10
5	0.5	60	10
6	0.5	70	10
7	1	80	10
8	1	90	10
9	2	100	10
10	3	110	10

## Appendix D

Simplification of canonical equation for two span cantilever beam with a fixed end and internal connection: *Fixed–Fixed–Free* Boundary:

Assuming all properties are constant along the length of the beam, this equation can be simplified to

(A1) $$EI\frac{\partial^{4}y\left(x,t\right)}{\partial x^{4}}+P\frac{\partial^{2}y\left(x,t\right)}{\partial x^{2}}-mr^{2}\frac{\partial^{2}\ddot{y}\left(x,t\right)}{\partial x^{2}}+m\ddot{y}\left(x,t\right)=f\left(x,t\right)$$

The equations of motion for both sub-spans are given by

(A2) $$EI\frac{\partial^{4}y_{1}\left(x_{1},t\right)}{\partial x_{1}^{4}}+P\frac{\partial^{2}y_{1}\left(x,t\right)}{\partial x_{1}^{2}}-mr^{2}\frac{\partial^{2}\ddot{y_{1}}\left(x,t\right)}{\partial x_{1}^{2}}+m\ddot{y_{1}}\left(x_{1},t\right)=f\left(x_{1},t\right),\;\;0\leq x_{1}\leq l_{1}\;EI\frac{\partial^{4}y_{2}\left(x_{2},t\right)}{\partial x_{2}^{4}}+P\frac{\partial^{2}y_{2}\left(x_{2},t\right)}{\partial x_{2}^{2}}-mr^{2}\frac{\partial^{2}\ddot{y_{2}}\left(x_{2},t\right)}{\partial x_{1}^{2}}+m\ddot{y_{2}}\left(x_{2},t\right)=f\left(x_{2},t\right),\;\;l_{1}\leq x_{2}\leq l_{2}$$

where $y_{1}\left(x_{1},t\right)$ and $y_{2}\left(x_{2},t\right)$ are the transverse displacements of the two sub-spans, while $x_{1}$ and $x_{2}$ are the spatial coordinates along the length of the sub beams at which the load is applied, measured from the fixed end. For the fixed end, at $x_{1}=0$, the moment and shear equilibriums are respectively given by

(A3) $$EI\frac{\partial^{2}y_{1}\left(0,t\right)}{\partial x_{1}^{2}}=0$$

(A4) $$EI\frac{\partial^{3}y_{1}\left(0,t\right)}{\partial x_{1}^{3}}+P\frac{\partial y_{1}\left(0,t\right)}{\partial x_{1}}-mr^{2}\frac{\partial^{2}\ddot{y_{1}}\left(0,t\right)}{\partial x_{1}^{2}}=0$$

The free end of the cantilever, $x_{2}=l_{2}$, allows rotational and vertical freedom. The moment and shear equilibriums respectively lead to

(A5) $$EI\frac{\partial^{2}y_{2}\left(l_{2},t\right)}{\partial x_{2}^{2}}+J\frac{\partial \ddot{y_{2}}\left(l_{2},t\right)}{\partial x_{2}}=0$$

(A6) $$EI\frac{\partial^{3}y_{2}\left(l_{2},t\right)}{\partial x_{2}^{3}}-M\ddot{y_{2}}\left(l_{2},t\right)-mr^{2}\frac{\partial^{2}\ddot{y_{2}}\left(l_{2},t\right)}{\partial x_{2}^{2}}=0$$

where M is the mass and J is the moment of inertia of the mass at the end of the beam.

An additional four boundary conditions are present at the internal fixed support for the moment equilibrium

(A7) $$EI\frac{\partial^{2}y_{1}\left(l_{1},t\right)}{\partial x_{1}^{2}}=0$$

(A8) $$EI\frac{\partial^{2}y_{2}\left(l_{1},t\right)}{\partial x_{2}^{2}}=0$$

(A9) $$EI\frac{\partial^{2}y_{1}\left(l_{1},t\right)}{\partial x_{1}^{2}}+P\frac{\partial y_{1}\left(l_{1},t\right)}{\partial x_{1}}-mr^{2}\frac{\partial^{2}\ddot{y_{1}}\left(l_{1},t\right)}{\partial x_{1}^{2}}=0$$

(A10) $$EI\frac{\partial^{2}y_{2}\left(l_{1},t\right)}{\partial x_{2}^{2}}+P\frac{\partial y_{2}\left(l_{1},t\right)}{\partial x_{2}}-mr^{2}\frac{\partial^{2}\ddot{y_{2}}\left(l_{1},t\right)}{\partial x_{2}^{2}}=0$$
